# Investigation of the response of tear-film neutrophils to interleukin 8 and their sensitivity to centrifugation, fixation, and incubation

**DOI:** 10.1038/s41598-020-75806-y

**Published:** 2020-11-12

**Authors:** Yutong Jin, Lyndon Jones, Maud Gorbet

**Affiliations:** 1 School of Optometry and Vision Science, University of Waterloo, Waterloo, Canada; 2 Department of Systems Design Engineering, University of Waterloo, Waterloo, Canada; 3 Centre for Ocular Research and Education, University of Waterloo, Waterloo, Canada

**Keywords:** Cell biology, Flow cytometry

## Abstract

During eye closure, a large number of neutrophils (polymorphonuclear neutrophils, PMNs) invade the ocular surface and are often referred to as tear-film PMNs. While immunophenotyping experiments have been performed on tear-film PMNs, the impact of commonly used experimental procedures on their phenotype as well as their response to interleukin-8 (IL-8), a physiological inflammatory mediator, have not yet been investigated. A gentle eye wash method was used to collect cells at home. In the morning upon awaking, participants washed their eyes with sterile phosphate buffer saline (PBS) and collected the runoff into a sterile polypropylene tube. The cell collection was then delivered to the lab within two hours. The effects of centrifugation, incubation and fixation with paraformaldehyde (PFA) before (pre-fixed staining) or after (post-fixed staining) incubation with antibodies were characterized. Tear-film PMNs as well as blood PMNs (used for comparison) were also stimulated with IL-8. To assess the reproducibility of cell collection and variability in receptor expression over time, participants were also asked to collect cells three times over a period of a month. The change in expression of surface receptors, CD11b, CD16, CD55, CD66b, important inflammatory and activation markers, and CD45 (PAN leukocyte marker) was assessed by flow cytometry. Fixing tear-film PMNs prior to the staining with antibodies resulted in a significant (fivefold or more) reduction in the expression of CD11b, CD16 and CD45 when compared to unfixed samples, while CD16 was the only receptor to undergo significant downregulation upon post-staining fixation. Furthermore, additional centrifugation step prior to antibody incubation as well as long (4 h) incubation at 37 °C resulted in significant reductions in expression of CD11b, CD16 and CD55 when compared to control samples. As opposed to blood PMNs, stimulating tear-film PMNs with IL-8 did not induce any significant changes in expression of CD11b, CD16, CD55 and CD66b. When working with collected tear-film PMNs, our results suggest that any additional centrifugation and incubation step should be avoided, or at least limited, and post fixation staining is recommended in order to preserve cell phenotype and cell integrity of tear film PMNs. Our study also adds further information on the reproducibility of the gentle eye wash as well as the inability of tear-film PMNs to modulate their surface receptors upon stimulation with IL-8. The latter may be due to prior exposure to IL-8, activation in the closed-eye environment, or a reduced ability to respond to inflammatory stimulus. Further mechanistic studies will be needed to gain a better understanding of the tear-film neutrophil phenotype.

## Introduction

Neutrophils (also known as polymorphonuclear neutrophils or PMNs) are essential inflammatory cells of the innate immune system^1^, and are the first cells to arrive at infected sites^2^. It is thought that, under normal physiological conditions, recruitment of immune cells to the ocular surface is limited due to immune privilege^3,4^. However, despite the immune privilege conferred to the eye, a significant infiltration of neutrophils occurs on the ocular surface when eyes close for a prolonged time (such as during sleep)^4–6^; these cells are often referred to as tear-film neutrophils^4^. Besides the presence of tear-film PMNs in the closed-eye environment, closed-eye tear fluid is also rich in inflammatory proteins, such as cytokines, lysozyme, lactoferrin, secretory IgA and complement products, suggesting a state of sub-clinical inflammation^6^.

Recent research indicates that the neutrophil population is heterogeneous, as PMNs found in different types of tissues, such as the lungs^7^, the mouth^8^, the placenta^9^ and the eye^4^, have been shown to exhibit distinct phenotypes. Tear-film PMNs have been reported to be quiescent upon stimulation and exhibit different expression of surface receptors associated with activation on their cell membranes in comparison to blood PMNs^4,10^. Although their origin is not established firmly, the hypothesis that they originated from the conjunctival blood vessels and entered the ocular surface through extravasation is supported by the low expression of L-selectin (CD62L) and high level of Mac-1 (CD11b)^4^. There are still many unknowns about their interaction with other immune cells, their extensive presence in the nocturnal environment and their functionality, that need to be further investigated.

PMNs express various types of surface receptors, such as cytokine receptors, G-protein coupled receptors, and Fc-receptors, which can induce different cell functional responses^11^. The level of receptor expression can also change upon activation and thus, evaluation of the expression of surface receptors provides information on cell’s activation state as well as a cell’s ability to respond to stimulus. Prior work has reported the response of tear-film neutrophils to phorbol-12-myristate-13-acetate (PMA; a potent synthetic chemical activator of protein kinase C), lipopolysaccharides (LPS; a bacterial endotoxin), *N*-formyl-L-methionyl- L-leucyl-L-phenylalanine (fMLP; a chemotactic peptide), and calcium ionophore^4, 10,12^. However, these are not all physiological stimuli nor are they representative of stimuli that tear-film PMNs would typically encounter in the closed-eye environment. Cytokines, such as tumor necrosis factor α (TNF-α)^13^, granulocyte–macrophage colony-stimulating factor (GM-CSF)^14^ and IL-8^15^ have been recognized to activate blood PMNs. IL-8, acting as a potent chemoattractant, can activate PMNs to induce several cell functional activities, such as regulating the transmigration from blood vessels to the inflammatory sites, respiratory burst, exocytosis and phagocytosis^16^. Corneal and conjunctival epithelial cells release IL-8^17^ and tears have been reported to contain various levels of IL-8 during the day and at night^18^. However, based on our knowledge, there has been no study on the response of tear-film PMNs to IL-8, a potent inflammatory mediator, which tear-film PMNs may be exposed to in the closed-eye environment. Furthermore, while changes in receptor expression have been reported in tear-film PMNs collected from the ocular surface^4,10^, limited information currently exists on how experimental procedures may affect expression of surface receptors.

Flow cytometry is often applied to study the expression of surface receptors on cells for diagnostic and phenotyping purposes as well as to characterize a response to inflammatory stimulus (cytokines, pathogens, foreign materials). In these studies, fixation, centrifugation and incubation are common steps used to process cells. Fixation can preserve cell structure, avoid undesired cell activation, prevent decomposition, putrefaction, autolysis and any artefactual changes^19,20^; whereas centrifugation can condense cells and allows excess antibodies and reagents to be removed.

Aldehydes, including paraformaldehyde (PFA) and formaldehyde^21^, are the most commonly used fixative agents and can preserve the cell structures by crosslinking the receptors and antibodies. PFA is the most commonly used fixative agent in flow cytometry, and the fixation process of blood cells, especially lymphocytes, has been thoroughly investigated. Cells can be fixed by either adding PFA before the staining with antibodies (pre-fixed staining) or adding PFA after the staining with antibodies (post-fixed staining). Pre-fixed staining has been shown to significantly decrease the expression of CD11b, CD18 and CD62L on blood leukocytes^22^. It has also been observed that expression of CD16 on blood PMNs gradually decreases with increased time of post-fixation^23^, suggesting that a fixation step can significantly affect the expression of the marker of activation being investigated. As heterogeneity exists in neutrophil population, it is not known if similar effects of PFA would be observed on the expression of membrane receptors on tear-film PMNs.

Neutrophils are also delicate and fragile cells that can be easily damaged by inappropriate experimental procedures^24^. For example, counterflow centrifugal elutriation, a protocol used to isolate PMNs, has been shown to cause an increased release of superoxide and granule contents when PMNs are activated^25^. Mechanical forces during centrifugation and resuspension as well as exposure to warm or cold temperature (37 °C vs. 4 °C) result in changes in the expression of C3b receptors (CD35) and CR3 (CD11b) on PMNs^26,27^. Since tear-film PMNs have a distinct phenotype from blood PMNs, it is likely unreasonable to assume that tear-film PMNs will be impacted by experimental procedures in the same manner as blood leukocytes.

Taken all together, experimental procedures have the potential to significantly affect results, and their impact on tear-film PMNs have been largely unexplored; it is a key component to ensure reproducibility and reliability of results. The objectives of this study were thus to determine if experimental procedures, such as centrifugation, incubation and fixation, could alter the expression of cell activation membrane receptors, to assess variation in receptor expression over a repeated collection and to investigate the response of tear-film PMNs to IL-8.

In this study, the expression of selected activation markers, namely CD11b, CD16, CD55, and CD66b, was examined on tear-film PMNs. These markers are commonly used to characterize the state of neutrophil activation and their ability to respond to inflammatory stimuli and contribute to the inflammatory response. These membrane receptors are also recognized to be important regulators of neutrophil effector functions in innate immunity. CD11b (also known as the integrin αM subunit of Mac-1) is upregulated upon activation. CD11b plays a crucial role in PMNs transmigration from the blood vessel to the site of inflammation and adhesion and also mediates several cell inflammatory responses^28^. CD16, a member of the Fc gamma receptors for immunoglobulin, has been shown to be associated with the phagocytosis, degranulation and apoptosis of PMNs^14,29^. CD55, a glycosylphosphatidylinositol (GPI)-anchored decay-accelerating factor (DAF), is one of the regulators of complement activation (inhibiting the activation of C3 and C5), can help to differentiate between bacterial and viral infection^30^ and which expression is modulated by inflammatory conditions^31^. CD66b is a GPI-anchored glycoprotein located in the specific granules; upon PMN activation and granule release, its surface expression increases, leading CD66b to also be referred to as a degranulation marker^32^. CD45 is a transmembrane protein tyrosine phosphatase expressed on leukocytes and is commonly used to identify leukocyte populations. CD45 upregulation has also been observed under certain inflammatory conditions and stimuli^33,34^. Changes in the expression of these surface receptors were evaluated by flow cytometry.

## Materials and methods

### Reagents and monoclonal antibodies

Fluorescein isothiocyanate (FITC)-conjugated monoclonal antibodies against human CD11b (clone: ICRF44) and CD66b (clone: G10F5), R-phycoerythrin (PE)-conjugated monoclonal antibodies against human CD55 (clone: IA10) and CD16 (clone: 3G8) and R-phycoerythrin-cytochrome 5 (PE-Cy5)-conjugated monoclonal antibody against CD45 (clone: HI30) were purchased from Becton Dickinson Pharmingen (San Diego, CA, USA). Paraformaldehyde (PFA), IL-8 human, Trypan Blue, Histopaque-1077 and PMA were from Sigma-Aldrich Co. (Oakville, Ontario, Canada). Phosphate-buffered saline (PBS) was purchased from Lonza (Allendale, New Jersey, USA). Dulbecco’s modified eagle media (DMEM) was purchased from Life Technologies (Burlington, Ontario, Canada). Keratinocyte serum-free medium (KM) was purchased from ScienCell (Carlsbad, California, USA).

### Collection of tear-film PMNs

This study was conducted in accordance with the tenets of the Declaration of Helsinki and received ethics clearance from the University of Waterloo Human Research Ethics Committee (#30164; Waterloo, ON, Canada). Informed consent was obtained from all participants. A total of nineteen healthy participants aged range from 23 to 33 were involved, and their ocular health was verified through slit lamp observations. Each participant provided one or more samples for each experiment.

Participants were provided with instructions and were trained to self-collect tear-film PMNs after sleep using a polypropylene pipette containing sterile PBS. They slept at home, as usual. After a full night of sleep (average 7 h), upon awakening, participants gently washed their eyes with approximately 5 mL of PBS for each eye, as demonstrated previously^4^; the eye wash was collected in one sterile polypropylene tube (pooled sample). After collection from both eyes, the tube was placed in the provided storage container and delivered to the lab within two hours. Cells were centrifuged at 280×*g* for 10 min at room temperature. Cell count and viability were determined by using trypan blue. Cells were diluted to a final cell concentration of 100,000 cells/mL in PBS if applicable.

### Collection of blood PMNs

Peripheral blood was drawn from three medication-free and healthy participants and added to a sterile polypropylene tube containing 5 U/mL of heparin (blood collection happened on separate days for each participant). After centrifugation at 100×*g* for 10 min, platelet-rich plasma was removed, and density-gradient centrifugation using Histopaque and Polymorphprep (Axis Shield PoC AS, Oslo, Norway) was performed on the blood sample. The isolated PMNs were washed three times with the first two washes in DMEM/10% FBS with 5 mM EDTA (to prevent any leukocyte activation) and the last wash was performed in sterile PBS. The purified blood PMNs were counted under the microscope with the hemocytometer (Hausser Scientific, USA), and their viability was determined by Trypan Blue. Cells were diluted to a final cell concentration of 100,000 cells/mL in PBS if applicable.

### Effect of fixation on tear-film PMNs (pre-fixed staining, post-fixed staining and no fixation)

For pre-fixed staining samples, a small aliquot of cell suspension was transferred into different labelled tubes containing 1:1 DMEM/10% FBS and 2% PFA (1% final concentration) followed by incubation at 4 °C for 15 min, the common protocol for pre-fixation of samples^22,35^. After fixing, fluorescently-labelled antibodies against CD11b, CD16 CD45, CD55, and CD66b were added to corresponding tubes and samples were incubated for 20 min in the dark. Following incubation with antibodies, samples were diluted by adding DMEM/10% FBS and stored at 4 °C until flow cytometry analysis the next day.

For post-fixation staining, the usual procedure for immunostaining was followed, whereby a small aliquot of cell suspension was incubated in tubes containing DMEM/10% FBS and fluorescently-labelled antibodies against CD11b, CD16, CD45, CD55 and CD66b. Samples were then incubated for 20 min at room temperature in the dark. At the end of incubation, samples were diluted with DMEM/10% FBS and fixed with PFA (1% final concentration). Samples were then stored at 4 °C until flow cytometry analysis the next day.

For the unfixed samples, the procedure remained the same as the post-fixation samples except in the last step, PFA was replaced by DMEM/10% FBS. The unfixed samples were analysed by flow cytometry within 15 min. Note that due to the effect of pre-fixation (see results), post-fixed staining method was used for the rest of the experiments.

### Effect of incubation and centrifugation on tear-film PMNs

Samples were diluted to a final cell concentration of 100,000 cells/mL in an artificial tear solution (keratocyte medium containing various tear film proteins) instead of PBS, as detailed in Table 1. KM is an endotoxin-free and sterile solution, containing various salts and amino acids and has previously been used as a tear substitute for in vitro studies.

**Table 1:** Components of the artificial tear solution in Keratinocyte medium^62^.

Components	Concentration (mg/ml)
Albumin	0.2
IgG	0.02
Lactoferrin	1.8
Lysozyme	1.9
Bovine Mucin	0.15

The osmolality of the artificial tear solution in KM was 295 ± 1 mmol/kg, as measured with the Wescor Vapro Osmometer, and is within the range of osmolality of normal tears^63^.

The non-incubated samples (“time zero”, 0 h sample, T0) were the controls, which were not incubated and processed directly after cell collection. To determine the impact of re-exposure to the physiological temperature of the closed-eye environment^36,37^, cell samples were incubated in polypropylene tubes for four hours in a cell incubator (37 °C, 5% CO_2_). These samples are referred to as “4-h incubated samples” (4 h samples).

Each 0 h and 4 h samples were further divided into centrifuged and non-centrifuged groups. Non-centrifuged groups stayed on the bench without any involvement of centrifugation, whereas centrifuged groups were spun down at 280×*g* for 5 min at room temperature. Then, cells were incubated with fluorescently-labelled antibodies against CD11b, CD16 CD45, CD55, and CD66b for 20 min in the dark at room temperature. Finally, samples were diluted with DMEM/10% FBS and fixed with PFA (1%, final concentration) and were assessed by flow cytometry after 24 h.

### IL-8 stimulation

Tear-film and blood PMNs were stimulated with IL-8 (10 ng/mL, final concentration) for 30 min at 37°C^38^. After stimulation, samples were stained with antibodies, fixed with PFA and analysed by flow cytometry after 24 h as described above.

### Flow cytometry

The PMNs population was identified by doubled-gating on cell size/granularity (forward-scattered light and side-scattered light, respectively) and CD45^+^ cells^4^. All samples were acquired on a Becton Dickinson FACS Calibur flow cytometer (Mountain View, CA, USA) using CELLQuest Software (Becton Dickinson, Mountain View, CA, USA). At least 2000 PMNs events were acquired, and fluorescent values for each antibody (also known as Mean Fluorescent Intensities, MFI) were recorded for all samples.

### Statistics

All results are reported as means ± standard deviations and are presented as the ratio of MFI of the experimental sample over the control sample. Statistical analysis was performed using a paired sample t-test or Wilcoxon signed-rank test. IBM SPSS Software (IBM Canada Ltd., Markham, Ontario, CA) was used, and a p-value of less than 0.05 was required for statistical difference. All graphs were plotted by GraphPad Prism Software (GraphPad Software, San Diego, CA, USA).

## Results

On average, the number of leukocytes collected by using the gentle eye wash method was 2.3 × 10^6^ with less than 2% dead cells (as determined by trypan blue).

### The impact of fixation

**Figure 1:**
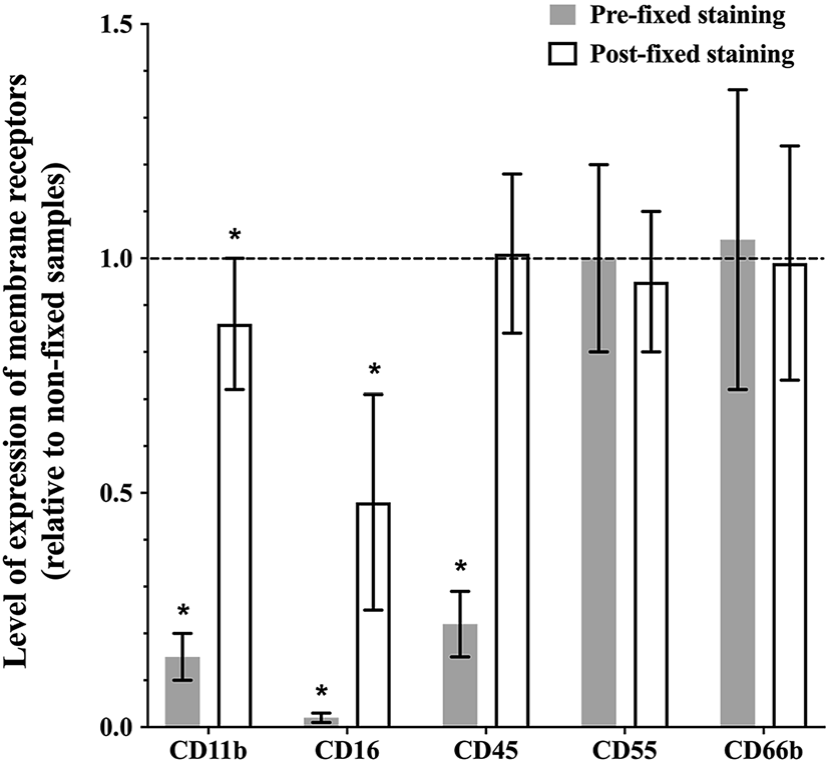
Effect of paraformaldehyde (PFA) on the expression of cell membrane receptors on tear-film PMNs. Cells were either fixed with 2% PFA at 4 °C for 15 min prior to staining with antibodies (pre-fixed staining) or stained with antibodies and then fixed with 2% PFA (post-fixed staining). Control samples were stained with antibodies and immediately analyzed by flow cytometry (non-fixed staining). All incubation with antibodies were performed at RT, in the dark, for 20 min. Fluorescence intensities for CD11b, CD16, CD45, CD66b, and CD55 were recorded as the arbitrary fluorescent units by flow cytometry and are expressed as a ratio of expression level on fixed samples compared to the control (non-fixed staining samples). The dotted line represents a ratio of 1 (no difference between fixed and control samples). Mean ± SD, n = 9, *significantly different from non-fixed samples, *p* < 0.01.

As shown in Fig. 1, pre-fixed and post-fixed staining affected the expression of activation markers on tear-film PMNs in different ways when compared to the non-fixed staining samples (control). When PFA was added prior to staining with antibodies, a significant decrease (78 to 98% reduction) was observed in the expression of CD11b (*p* < 0.001), CD16 (*p* = 0.008), and CD45 (*p* < 0.001). Conversely, the expression of CD55 and CD66b remained relatively unchanged (*p* ≥ 0.84). On the other hand, in the post-fixed staining group, a reduction of more than 50% was observed in the expression of CD16 (*p* = 0.008) and a small (14%) but statistically significant reduction in expression of CD11b (*p* = 0.01). The other markers, CD45, CD55 and CD66b, on post-fixed staining samples displayed some variations in expression but remained relatively unchanged (*p* ≥ 0.36). The results indicated that exposing collected cells to PFA prior to staining with antibodies resulted in drastic reductions in CD11b, CD16 and CD45 expression and thus for the remainder of the study, cells were fixed post staining.

### The impact of incubation temperature

**Figure 2:**
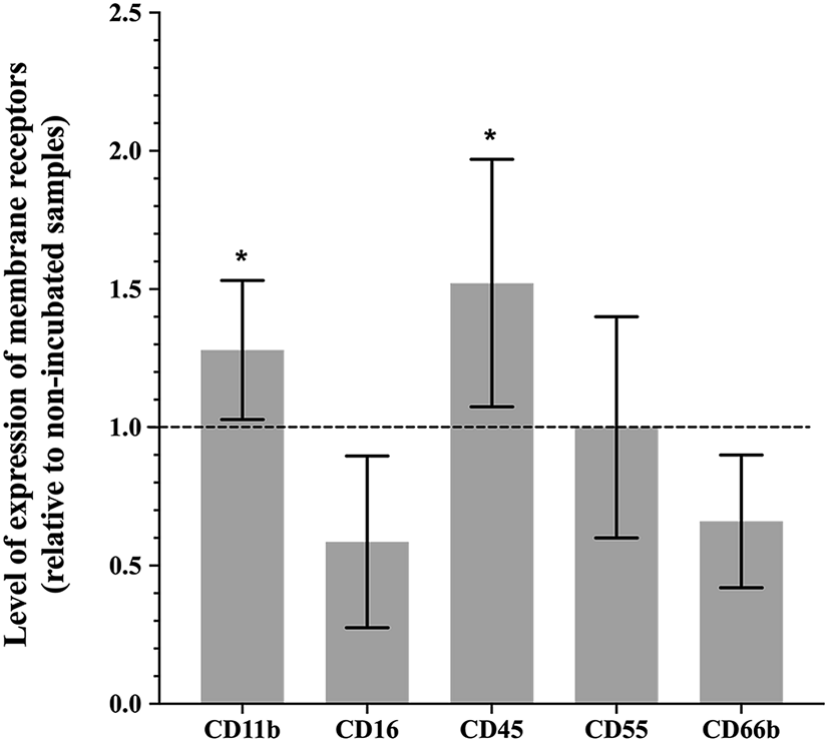
Effect of exposure to 37 °C for 4 h on the expression of cell membrane receptors on tear-film PMNs. Following collection, tear film PMNs were either processed for flow cytometry (antibody staining followed by PFA fixing) or incubated for 4 h at 37 °C, 5% CO_2_. Following 4 h incubation, cells were stained with antibodies and fixed with PFA. Fluorescence intensities for CD11b, CD16, CD45, CD66b, and CD55 were recorded as arbitrary fluorescent units by flow cytometry and are expressed as the ratio of expression level on incubated samples compared to the control (non-incubated samples, 0 h sample). The dotted line represents a ratio of 1 (no difference between fixed and control samples). Mean ± SD, n = 8, *significantly different from non-incubated samples, *p* ≤ 0.04.

To assess how exposure to physiological temperature following collection may activate tear-film PMNs, cells were exposed to a 4-h incubation at 37 °C, 5% CO_2_. Changes in expression of cell activation membrane receptors are reported in Fig. 2. When compared to the 0 h sample (control), exposing collected tear film PMNs to 37 °C for 4 h appeared to induce a mild state of cell activation, with a significant (*p* ≤ 0.04) upregulation in CD11b (~ 30% increase) and CD45 (~ 50% increase) while expression of CD16 and CD66b decreased markedly; however, due large variations in response, the more than 30% reduction in CD16 and CD66b expressions were not statically significant (*p* = 0.09 and *p* = 0.06, respectively). The reduction in CD66b was inconsistent with the lower expression of CD16, an indicator of degranulation that is cleaved upon degranulation, as CD66b expression is expected to increase with release of granules. CD55 expression remained mostly unchanged (*p* = 0.78).

### The impact of centrifugation

**Figure 3:**
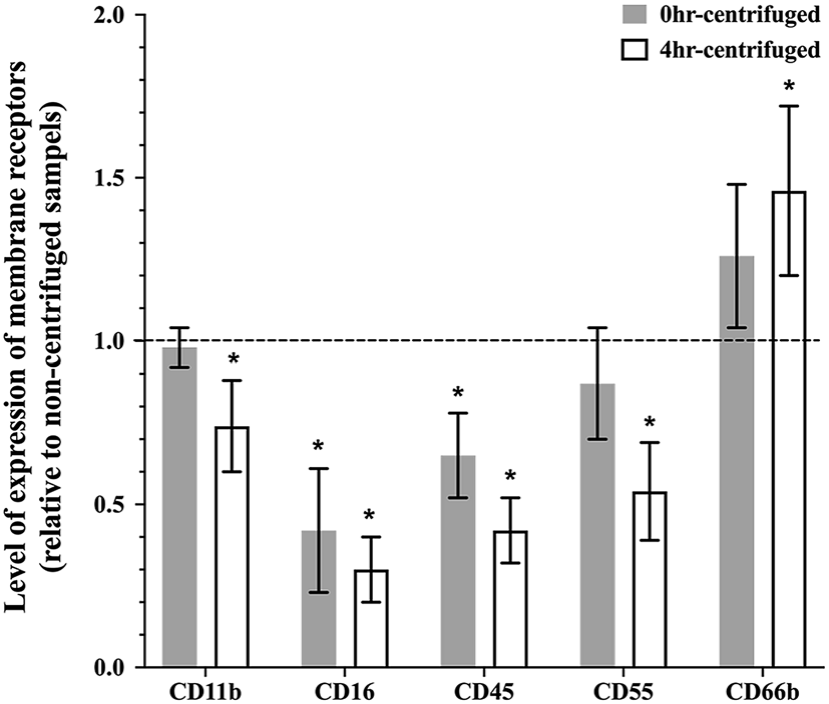
Effect of centrifugation on the expression of cell membrane receptors on tear-film PMNs. Following collection and concentration, tear film PMNs were either subjected to another centrifugation step or incubated for 4 h at 37 °C–5%CO_2_ and then centrifuged. Samples were processed for flow cytometry (antibody staining followed by PFA fixing). Fluorescence intensities for CD11b, CD16, CD45, CD66b, and CD55 were recorded as arbitrary fluorescent units by flow cytometry and are expressed as percentage of expression level on centrifuged samples compared to controls (non-centrifuged samples). The dotted line represents a ratio of 1 (no difference between centrifuged and non-centrifuged samples). Mean ± SD, n = 8–10, *significantly different from control, *p* ≤ 0.03.

To determine the effect of centrifugation on the expression of cell membrane activation markers, tear-film PMNs from 0 and 4 h-incubated groups were either centrifuged at 280×*g* (5 min) or not. Changes in the expression of cell activation membrane receptors are reported in Fig. 3. For 0 h-centrifuged samples, results indicated that an extra centrifugation step, which exposed cells to mechanical forces, caused some degranulation, as a significant decrease in the expression of CD16 (*p* < 0.001) and an increase in CD66b (*p* = 0.16) were observed. Reduction in the expression of CD45 (*p* < 0.001) and CD55 (*p* = 0.07) also occurred. The expression of CD11b was not affected by centrifugation (*p* = 0.22).

Comparing the 0- and 4 h-centrifuged samples, all changes observed with the 0 h-centrifuged samples were further amplified after the 4 h incubation at 37 °C, confirming our above results that cells were affected by the 37 °C incubation and that exposing cells to centrifugal mechanical forces induces further cell damage and degranulation. The expressions of CD11b (*p* = 0.03), CD16 (*p* = 0.003), CD45 (*p* < 0.001) and CD55 (*p* = 0.004) were significantly reduced while, as expected from a degranulation process, the levels of CD66b (*p* < 0.001) significantly increased.

### Variations in phenotype of tear-film PMNs between collection days

**Figure 4:**
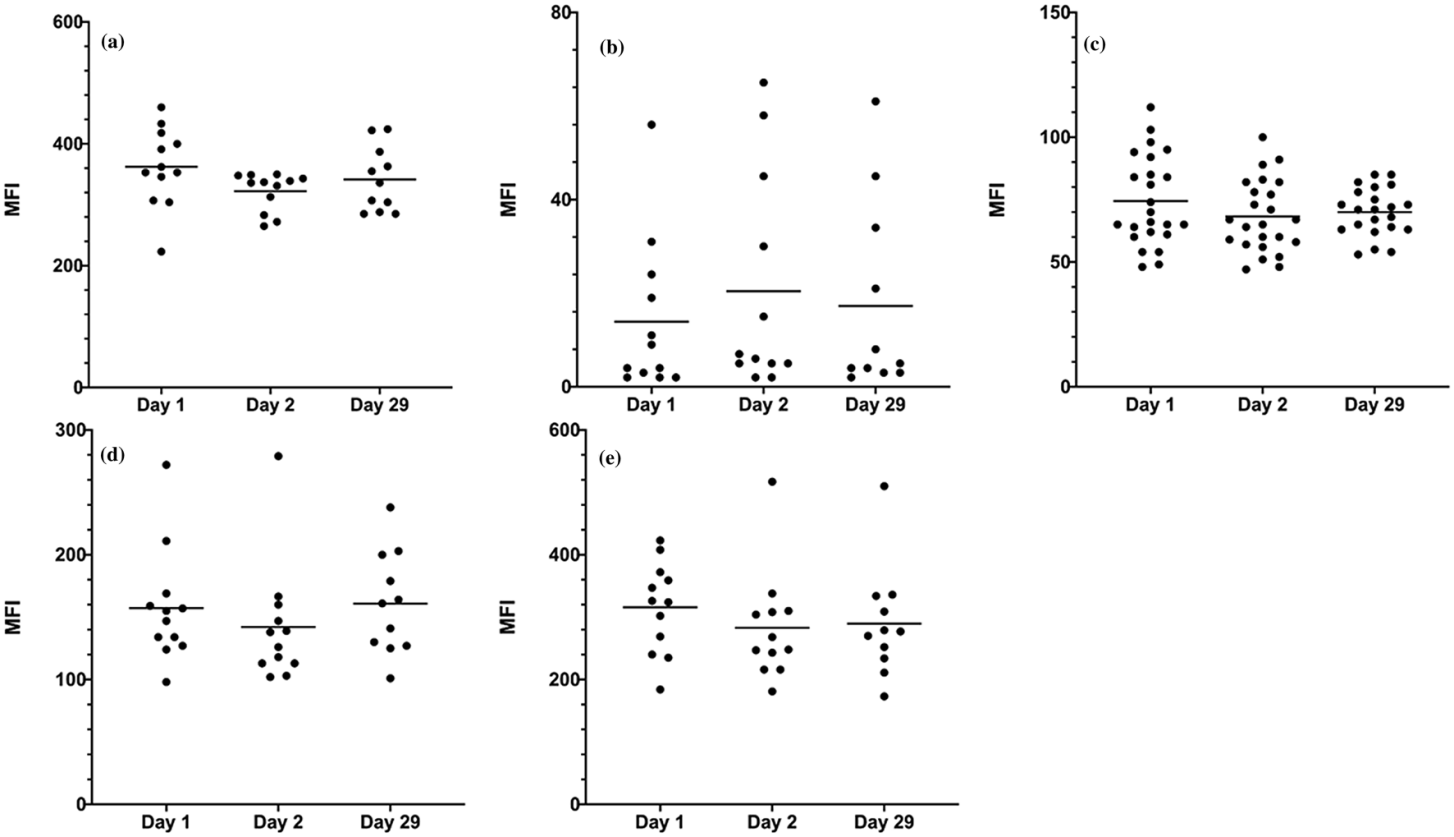
Variations in the level of expression of membrane receptors on tear-film PMNs collected over 3 collection days (MFI: Mean Fluorescent Intensity). (**a**) CD11b; (**b**) CD16; (**c**) CD45; (**d**) CD55; (**e**) CD66b. n = 12. Twelve participants collected cells on day 1, day 2 and day 29 (one participant did not have enough cells on day 29). Note that (**c**) shows duplicates as CD45 expression on PMNs is measured used in 2 combination of antibodies (CD11/CD16/CD45 and CD66b/CD55/CD45).

Twelve participants provided a cell collection sample three times in a month (day 1, day 2 and day 29) to assess variations in the phenotype of collected tear-film PMNs. All samples were acquired using the same flow cytometer settings and thus difference in fluorescence intensity (MFI) represents a difference in receptor expression. As reported in Fig. 4, the level of expression of membrane receptors on tear-film PMNs remained constant across the collection days, with a small non-statistically significant decrease in CD11b, CD45, CD55 and CD66b expression between day 1 and day 2. While changes in level of expression of CD16 across collection days did not reach statistical significance, large variability in CD16 expression was observed not only across days but within single participant collection data, where for example on day 1, a MFI of 56 would be recorded, 5 on day 2 and then 34 on day 29.

The level of expression of the membrane receptors on tear-film PMNs after collection was also compared to that of blood PMNs. As shown by the MFI values in Table 2, expression of CD11b, CD55 and CD66b on freshly collected tear-film PMNs was significantly higher than that of blood PMNs, and CD16 expression was significantly lower on tear-film PMNs (*p* < 0.001). Of note also is the high standard deviation observed in the expression of CD16 on tear film PMNs: with some participants’ PMNs exhibiting “background level”/low fluorescent intensity (with a MFI in the order of 3) while in others, a MFI as high as 120 could be recorded.

**Table 2:** Expression of selected membrane receptors on unstimulated tear-film and blood PMNs. Values are reported as mean fluorescent intensities (MFI).

CD markers	Tear-film PMNs	Blood PMNs
CD11b	358 ± 79*	48 ± 22
CD16	28 ± 31*	841 ± 198
CD45	71 ± 14*	47 ± 17
CD55	154 ± 44*	55 ± 26
CD66b	297 ± 83*	35 ± 22

Results are presented as means ± SD from 12 participants collecting tear-film PMNs several times (n = 53) and 3 participants donating the blood PMNs (n = 6).

*Significantly different from blood PMNs (*p* < 0.001).

### Responses of tear-film and blood PMNs to IL-8

**Figure 5:**
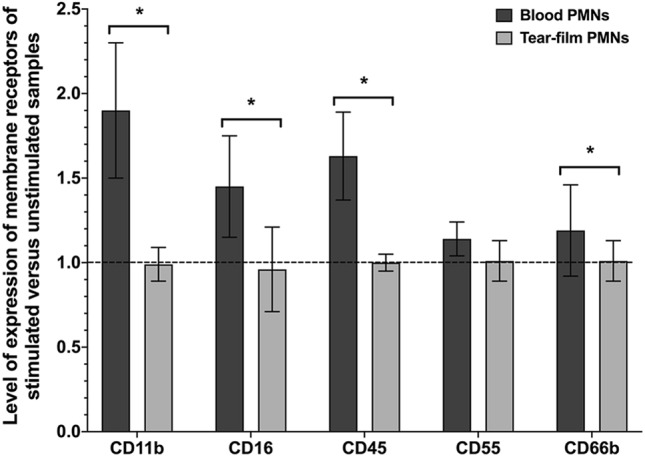
The expressions of surface receptors of blood PMNs and tear-film PMNs following stimulation with IL-8. PMNs were stimulated with IL-8 (10 ng/mL), and their expression of surface receptor of CD11b, CD16, CD45, CD55 and CD66b was assessed through flow cytometry. Data is expressed as the ratio between stimulated (IL-8) and unstimulated samples. The dotted line represents the unstimulated value. Results are presented as means ± standard deviations from 13 participants collecting tear-film PMNs several times (n = 30) and 3 participants donating blood at least once (n = 4). *Significantly different from blood PMNs (*p* < 0.03).

After stimulation with IL-8 (10 ng/ml, 30 min at 37 °C), there was no change in surface receptor expression in tear-film PMNs when compared to unstimulated samples (control) (*p* = 0.12), as shown in Fig. 5. On the other hand, in blood PMNs, IL-8 induced a drastic increase in the expressions of CD11b, CD16 and CD45, while the levels of CD55 and CD66b remained relatively unchanged.

## Discussion

In immunophenotyping studies, it is crucial to ensure that experimental procedures before labelling with fluorochrome-conjugated antibodies exert a minimal effect on antigen expression, and all the more so when novel cell populations, such as tear-film PMNs and their response to inflammatory stimuli, pathogens or biomaterials, are being characterized. Our study investigated the effect of pre-fixation and post-fixation staining, incubation, and centrifugation on the expression of membrane receptors on tear-film PMNs to optimize experimental procedures and preserve the expression of surface receptors as close as possible to the in vivo state by limiting in vitro changes. Any significant change induced by procedures would significantly impact our interpretation of the results on the activation state of tear-film PMNs and their possible role on ocular inflammation.

Fixation aims to preserve the binding structure for a prolonged time by crosslinking antigens with their antibodies. However, research has shown that exposure of cells to fixative chemicals might cause damage to the expressions of function-associated antigens^39^, and an increase in autofluorescence^40^. According to our results, pre-fixed staining significantly decreased the expressions of CD11b (an adhesion and activation marker), CD16 (a phagocytosis and degranulation marker) and CD45 (a PAN leukocyte marker) on tear-film PMNs. A decrease in the expression of CD11b with fixation prior to staining has also been reported with blood PMNs and hematopoietic cells^35,39,40^. Fixation prior to staining may lead to a loss of antigenicity of the epitopes^41^. Aldehyde fixative reacts with amine groups and forms a methylene bridge to crosslink between proteins; during this process, the fixative may distort the tertiary structure of proteins^35^, and consequently, cause the decrease in recognition of antibodies to their corresponding antigens. Depending on their structure, antigens may be affected differently which would explain why a pre-fixation step induced significant damage on CD11b, CD16 and CD45 but not on CD55 (a complement activation marker) and CD66b (a degranulation marker). The changes induced by exposure to PFA prior to staining would have a significant impact on our interpretation of results, i.e. low CD16 expression would indicate that tear film neutrophils had undergone extensive degranulation in vivo, although in this case, this would be an artefact of how the cells were processed following collection.

A fixation step following staining significantly reduced the expression of CD16 and CD11b. Although the 14% downregulation of CD11b compared to the non-fixed sample was statistically significant, it may not carry biological significance and, in this case, would not significantly affect our interpretation of results in the context of cell activation. On the other hand, as with pre-fixed staining, the change observed with CD16 warrants caution. A dramatic decrease in the expression of CD16 was previously reported by Stewart et al. after 24 h fixation on blood PMNs, and expression was also observed to further decrease between 24 and 96 h after fixation^23^. They also observed a small but significant reduction (12%) in CD11b after 24 h fixation^23^, which is closed to the 14% reduction in CD11b we observed in our post-fixed samples analysed around 24 h. Interestingly, we also observed a change in levels of expression with some of our activation markers depending on the time of storage after fixation post staining (results not shown), which is in agreement with Stewart et al*.*’s research on blood PMNs^23^. Thus, to increase result reproducibility, all samples were always analyzed around 24 h after fixation. The decrease in level of expression during 4 °C storage after fixation may be due to internalization of surface receptors, as fixation tends to permeabilize cell membranes, resulting in a decrease in cell membrane integrity, which could eventually cause membrane receptors to be internalized^23,35^. The fact that not all receptors are affected the same way is not surprising, as differences exist in the stability of the antigen and antibody bond and the linkage between the antibody and its fluorochrome^42^. Fine et al. has also reported that PMNs are sensitive to fixation protocols. In their studies on oral PMNs, a small increase in CD11b and CD18 as well as a small decrease in CD63 were observed with post fixing, while levels of CD66a remained unchanged^43^. The changes reported appear minimal compared to our results with tear-film PMNs (i.e. significant reduction in CD11b and CD16 with pre-fixing), further highlighting the importance of not extrapolating results between PMN populations^43^.

Tear-film PMNs incubated at 37 °C for 4 h showed a significant upregulation in CD11b and CD45, suggesting activation of tear film PMNs, which would be agree with previous observation in blood leukocytes^44,45^. This upregulation is noteworthy as CD11b upregulation on tear-film PMNs has been shown to be minimal upon stimulation with fMLP, PMA, and LPS when collected after sleep^4^. Thus, the change in temperature may be a potential stress or stimulus to activate tear-film PMNs. However, not all results support the activation of tear-film PMNs during the 37 °C incubation, as a decrease in CD66b was also observed (activation associated with degranulation would have resulted in an increase in CD66b expression). Since the downregulation of CD66b was also associated with lower levels of CD16, this may also be indicative of apoptosis^14^. Large variations observed in 4 h-incubated samples further suggested that tear-film PMNs may not remain stable after 4 h incubation. Similar observations have been made with blood leukocytes where in vitro experiments tend to be limited to 2 h to preserve cell integrity^46^. The incubation of tear-film PMNs in round-bottom tubes, which may cause cell clumping, may also have contributed to the observed alterations in surface receptor expression.

Our study showed that centrifugation resulted in a downregulation of all our selected cell membrane receptors except CD66b. Centrifugation is a necessary initial first step to concentrate cells, but additional centrifugation steps are often performed to wash cells, re-concentrate cells, etc. Since the centrifugation step was performed before immunostaining, the reduced expression cannot be explained by the antibodies being washed away. During centrifugation, cells are precipitated to the bottom of the tube and form a pellet. The changes in the expression of surface receptors may be due to cell–cell interaction in the pellet or contact with the internal biomaterial (polypropylene) surface. The observed downregulation in receptor expression may also be due to the internalization of surface receptors by tear-film PMNs due to damaged cell membrane integrity induced by exposure to the sheer force of the centrifugation. This would be corroborated by the fact that centrifugation of the 0 h samples had a lesser impact on the expression of surface receptors than in samples incubated for 4 h: centrifugation may induce some damage to cell integrity, which is further exacerbated in the 4 h-incubated sample which may undergo activation and apoptosis (see above) due to the 4 h incubation at 37 °C. Interestingly, previous work with blood isolated leukocytes have reported either an increase in receptor expression or no change following washing steps^47,48^. It is also possible that the changes in the expression of surface receptors during centrifugation is an indication of potential degranulation during this step. Four types of granules exist in PMNs, the azurophil or primary granules, the specific or secondary granules, the gelatinase granules, and secretory vesicles^49^. Upon the release of azurophil granules, the expression of CD63, CD68 is upregulated, and neutrophil elastase is released^50^. CD66b expression increases upon release of specific granules, while PMNs upregulate expression of CD14, CD16 and CD55 upon release of secretory vesicles^50–52^. The downregulation of CD16 upon activation has been shown to be associated with the cleavage by proteases, specifically neutrophil elastase^10,53^. Therefore, the increase in CD66b expression and decrease in CD16 expression in the 4 h-centrifuged samples may suggest the release of primary granules containing neutrophil elastase which then cleaved CD16. These points further highlight how centrifugation steps have the potential to affect receptor expression in various ways.

IL-8 has been shown to cause an increase in the expression of Mac-1 (CD11b/CD18) to promote adhesion of PMNs^54,55^. In our study, as expected, IL-8 induced cell activation in blood PMNs. However, no change was observed in tear-film PMNs upon stimulation with IL-8. Almost 95% of Mac-1 is stored in cytoplasmic granules and can be rapidly mobilized to the PMNs cell surface during/after activation^2,52^. Hence, the impaired upregulation of CD11b of tear-film PMNs may be caused by the shortage of cytoplasmic stores, as tear-film PMNs already show a high level of CD11b expression (see Table 2). Thus, the observed lack of response to IL-8 may further corroborate previous observations by Postnikoff et al. and the hypothesis that tear-film PMNs collected after sleep have been primed or are in an activated state and unable to respond further^10,12,56^.

Gorbet et al*.* showed that tear-film PMNs were unable to respond to fMLP^4^. The fMLP and IL-8 receptors (IL-8Rs) are members of the seven-transmembrane spanning receptor (STMR) coupled to a G protein^57^, so IL-8 and fMLP may have some similarities in the signal transduction pathways^58^. The inability for tear-film PMNs to respond to IL-8 may occur because of the same impairment within the pathways with fMLP or/and the receptors^58,59^. Downregulation of IL-8Rs may also have caused the lack of response to IL-8 of tear-film PMNs, as exposure to IL-8 has been suggested to induce internalization of IL-8Rs in the sputum of asthma patients^60^. As high concentrations of IL-8 have been reported in the closed-eye environment^12,61^, further studies will be required to explore this hypothesis since the expression of IL-8Rs on tear-film PMNs has not been investigated so far.

As discussed above, various steps in the experimental procedures were shown to have an impact on receptor expression. After the flow cytometry protocol was optimized, participants were recruited to perform repeated collection to assess the potential for variation within individuals. Small changes occurred in the level of expression of the membrane receptors on the collected cells, except for CD16 where high variability was observed inter- and intra-individuals. The changes observed in CD16 expression may be due to underlying closed-eye conditions, as its downregulation is correlated with elastase release. The variability in CD16 expression would suggest that this membrane receptor may be more difficult to use as indicator for future diagnostic and phenotyping purposes. Collectively, the intra difference (within a single subject) expression of CD11b, CD45, CD55 and CD66b was relatively small, suggesting that these markers may be used more reliably with one cell collection to characterize cell phenotype and inflammatory potential.

## Conclusions

This study demonstrates that expression of membrane receptors on tear-film PMNs exhibit different sensitivity to fixation, centrifugation and incubation, and thus caution should be exerted to ensure that experimental procedures will not affect markers of cell activation as this has the potential to significantly impact result interpretation. Due to the drastic decrease in expression of CD11b, CD16 and CD45, it is recommended to fix cells after staining with antibodies to minimize changes in activation markers. Additional centrifugation step after the initial concentration/wash as well as long exposure to 37 °C (4 h or more) of tear-film PMNs induce cell damage or activation, and thus should be minimized unless appropriate controls are in place. Since variations in CD16 were observed across different days of cell collection for each participant, it may be difficult to use CD16 for diagnostic purposes. Furthermore, tear film PMNs collected from the closed eye environment were shown to be unable to up or down regulate cell activation membrane receptors upon IL-8 stimulation, suggesting an impairment that may be explained by prior exposure to IL-8 and/or activation in the closed-eye environment. This study contributes significant new knowledge on the effect of experimental procedures and the phenotype of tear-film PMNs. The results presented in this study will enable to more reliably characterize tear film PMNs and gain a better understanding of their phenotype and their role in ocular surface inflammation.

## Data Availability

The datasets analysed during the current study are available from the corresponding author upon reasonable request.
